# C-Reactive Protein as a Biomarker for Major Depressive Disorder?

**DOI:** 10.3390/ijms23031616

**Published:** 2022-01-30

**Authors:** Laura Orsolini, Simone Pompili, Silvia Tempia Valenta, Virginio Salvi, Umberto Volpe

**Affiliations:** Unit of Clinical Psychiatry, Department of Neurosciences/DIMSC, Polytechnic University of Marche, 60126 Ancona, Italy; l.orsolini@staff.univpm.it (L.O.); smn.pmpl@gmail.com (S.P.); silvia.tempia@gmail.com (S.T.V.); v.salvi@staff.univpm.it (V.S.)

**Keywords:** CRP, C-reactive protein, depression, inflammation, major depressive disorder, neuroinflammation

## Abstract

The etiopathogenesis of depression is not entirely understood. Several studies have investigated the role of inflammation in major depressive disorder. The present work aims to review the literature on the association between C-Reactive Protein (CRP) and depression. A systematic review was performed for the topics of ‘CRP’ and ‘depression’ using the PubMed database from inception to December 2021. Fifty-six studies were identified and included in the review. Evidence suggested the presence of dysregulation in the inflammation system in individuals with depression. In most studies, higher blood CRP levels were associated with greater symptom severity, a specific pattern of depressive symptoms, and a worse response to treatment. Moreover, about one-third of depressed patients showed a low-grade inflammatory state, suggesting the presence of a different major depressive disorder (MDD) subgroup with a distinct etiopathogenesis, clinical course, treatment response, and prognosis, which could benefit from monitoring of CRP levels and might potentially respond to anti-inflammatory treatments. This work provides robust evidence about the potential role of CRP and its blood levels in depressive disorders. These findings can be relevant to developing new therapeutic strategies and better understanding if CRP may be considered a valuable biomarker for depression.

## 1. Introduction

Depression is the most common mental illness affecting around 10–20% of the general population [1]. In 2008, the World Health Organization (WHO) ranked depression as the third cause of burden of disease worldwide and projected that it will rank first by 2030 [2]. Depression also represents the major contributor to suicide deaths, with an incidence of up to 800.000 per year worldwide [3]. It usually manifests in early adulthood, with a mean age onset of approximately 20–25 years [4]. Depression usually occurs 2-fold higher among women than men at all age groups, and several socio-demographic risk factors are implicated in this trend [5,6]. However, the etiopathogenesis of depression is highly complex and not entirely understood. One of the most widely renowned hypotheses is based on the monoaminergic theory [7], although recent researches focused on other pathways, such as the dysregulation of hypothalamus-pituitary-adrenal axis (HPA), genetic susceptibility, and epigenetic modifications, the oxidative stress-induced damage, and the neurodevelopment theory [8,9,10,11,12].

Furthermore, recent evidence suggested a possible role of immune dysregulation in the etiopathogenesis of depression [13,14,15,16]. Accordingly, it has been well documented that individual affected with autoimmune disorders (e.g., rheumatoid arthritis, psoriasis, diabetes mellitus, chronic inflammatory bowel disease, and autoimmune thyroiditis) are more likely to display comorbid depressive symptomatology [17,18,19,20,21,22,23]. Likewise, depression is often associated with other conditions where an increased inflammatory state has been documented, such as in cardiovascular diseases, obesity, smoking status, and specific nutritional deficits [24,25,26]. Furthermore, there is strong evidence that the activation of the innate immune system may lead to “sickness behaviors” characterized by depression-like symptomatology, including anhedonia, weight and appetite loss, memory impairment, as well as cognitive and social dysfunction [27,28].

Furthermore, several studies investigated the role of a set of specific inflammatory biomarkers in depressive disorders, including C-Reactive Protein (CRP) [15,16,29]. CRP is a pentameric acute phase reactant protein secreted mainly by hepatocytes in response to the activation of the innate humoral system [30]. It is easily measured through a blood sample also in its high-sensitivity form (hs-CRP) [31] and used in clinical practice mainly as a biomarker of infection, chronic disease state, and chronic low-grade inflammation [30,32]. CRP usually does not freely cross BBB [33], even though several mechanisms have been hypothesized to explain how CRP can interact with the Central Nervous System (CNS) [33,34]. For instance, it has been suggested that an increase of the blood-brain barrier (BBB) permeability could be determined by severe stress and/or traumatic brain injury [35,36]. Furthermore, neurovascular damage induced by the peripheral myeloid cells, pro-inflammatory cytokines, and the dysregulation of the complement pathways could be a further potential mechanism of action [36,37,38]. In addition, CRP appears to determine BBB disruption, through the binding with some ligands (i.e., Fc gamma receptors, CD16, CD32), expressed in the microglia, astrocytes, and endothelial cells [39]. Moreover, increased levels of CRP and its proinflammatory activity can drive CNS inflammation through microglia and astrocytes activation [40,41,42].

Therefore, the present review aims at systematically investigating the role of CRP in depressive disorders, including Major Depressive Disorder (MDD) and Treatment-Resistant Depression (TRD) in order to better clinically characterize depressed patients, also taking into account the neuroinflammatory state. A secondary aim is evaluating whether CRP may represent a useful biomarker in clinical practice, able to early identify and characterize those depressed patients according to their different illness stages, severity and/or treatment resistance. Moreover, a third aim is at evaluating whether a low- and/or medium neuroinflammatory state may predict different treatment responses and, hence, address clinicians towards a tailored treatment.

## 2. Material and Methods

### 2.1. Search Sources and Strategies

This systematic review was performed according to the guidelines recommended by Cochrane Collaboration [43] and the Preferred Reporting Items for Systematic Reviews and Meta-Analyses (PRISMA) [44]. Literature searches were conducted by using PubMed database, from its inception to 10 December 2021, using the following keywords: ((((“*C-reactive protein*” [Title/Abstract]) OR (*CRP* [Title/Abstract])) OR (“*hs-CRP*” [Title/Abstract])) OR (*hsCRP* [Title/Abstract])) AND (*depressi* * [Title/Abstract]).

### 2.2. Study Selection

All studies evaluating the relationship between CRP and depression/depressive disorders/MDD/TRD, based only on human participants and published in English, were screened. Firstly, all papers were screened according to their titles and abstracts and full tests of relevant studies were obtained. Narrative reviews, systematic reviews, meta-analyses, letters to the editor, book chapters, case-reports, case-series, and duplicates were excluded. The following inclusion criteria were considered to be included in the present review: (a) studies investigating CRP levels in depressive/MDD/TRD patients; (b) studies assessing the depressive symptomatology through validated semi-structured clinical interviews and/or rating scales. Exclusion criteria include: (a) studies investigating other inflammatory proteins (e.g., interleukins, fibrinogen), hormones (e.g., cortisol, thyroxine) or other types of biomarkers, even though on sample constituted by depressed/MDD/TRD patients; (b) studies in which depression is comorbid with other organic pathologies (e.g., cancer, rheumatoid arthritis, diabetes mellitus) and/or psychiatric disorders (e.g., schizophrenia, bipolar disorder); (c) in vitro or animal studies.

### 2.3. Data Extraction and Management

Independently, two authors (S.P. and S.T.V.) read all included full-text papers, by using the abovementioned inclusion and exclusion criteria. All relevant screened papers to be included were collected and data were extracted in a dedicated excel spreadsheet. The disagreement was resolved by discussion and consensus with a third member of the team (L.O.).

### 2.4. Characteristics of Included Studies

**Figure 1:**
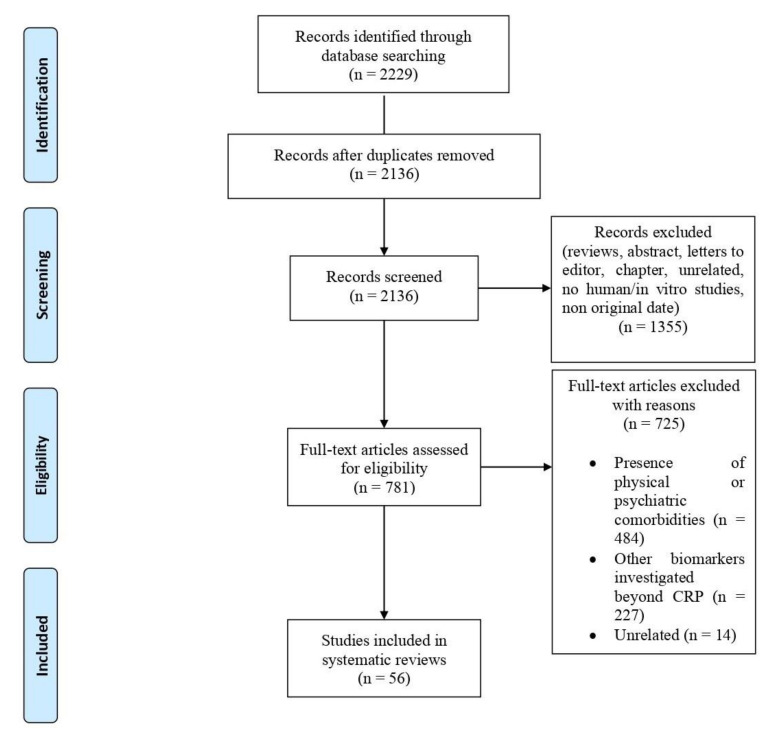
PRISMA Flow Diagram [44].

Literature search turned up 2229 results, out of which 97 met the inclusion criteria and were included in our analysis (Figure 1). Table 1 summarizes the main characteristics (study design, sample size, main outcomes, and findings) of the studies included here.

### 2.5. Quality Assessment

SP and STV independently assessed the quality of included studies using the modified versions of the Newcastle-Ottawa Quality Assessment Scale, adopted for cross-sectional (Table 2), case-control (Table 3), cohort studies (Table 4) and RCT (Table 5) [99]. The scale investigated the selection, comparability, and the main outcomes of the studies. A score of 7 or above is considered for a good quality; a score of 5–6 indicate a satisfactory quality level and scores less than 5 usually indicate unsatisfactory studies (Supplementary Material: PRISMA_2020_checklist). Eventually, disagreement was resolved by L.O.

## 3. Results

### 3.1. Studies on the Association between CRP Levels and Depression

#### 3.1.1. Cross-Sectional Studies

A cross-sectional population-based study evaluated the association between depression and CRP in 6126 adults, assessed with the Center for Epidemiologic Studies Depression (CESD) scale [70]. Subjects with depressive symptoms (CESD ≥ 16) displayed on average a CRP concentration of 0.43 mg/L (95% CI = 0.16–0.72) higher than the healthy controls. In addition, the authors found that this association appeared to persist, also when the sample size was limited only to depressed patients without any chronic disease. A large population-based cross-sectional study recruited 5447 Korean people aged > 20 years, from the Korean National Health and Nutrition Examination Survey (KNHANES VII-1), to assess the association between hs-CRP and depression [61]. High hs-CRP levels were defined as ≥ 3.0 mg/L and depression evaluated through the Patient Health Questionnaire-9 (PHQ-9). Individuals with high CRP levels had a significantly higher rate of depression compared to the others. In addition, hs-CRP levels were independently associated with PHQ-9 scores, even after adjustment of confounders. Furthermore, the authors found that this association appeared to be more prominent in younger adults than in older patients [61]. Using the abovementioned nationwide cross-sectional survey (i.e., KNHANES), Cho et al. (2021) [52] confirmed in a sample of 10,702 adults, that individuals with high hs-CRP levels (>3.0 mg/L) were more likely to have depressive symptomatology and suicidal ideation compared to those participants with low hs-CRP concentrations. In the subgroups analysis, the association was more prominent in the non-geriatric subsample (i.e., ≤64 years), in males, in obese adults, and in those who did not report regular aerobic physical activity.

A US-population-based study coming from the National Health and Nutrition Examination Survey (NHANES), recruited 16 patients with atypical MDD, 93 with nonatypical MDD and 1682 healthy individuals, to measure CRP levels [58]. Subjects with atypical MDD exhibited higher CRP levels than those subjects without MDD (mean difference = 1.56 mg/L) or nonatypical MDD (mean difference = 1.40 mg/L) control, even after adjusting for confounders, anxiety disorders, BMI, and smoking [47]. A recent retrospective, observational, cross-sectional study evaluated the differences between elevated CRP levels (i.e., CRP > 3 mg/L and ≤ 10 mg/L) in subjects with MDD, bipolar disorder (BD), and obsessive-compulsive disorder (OCD) [47]. The authors recruited 388 patients (156 MDD, 135 BD and 97 OCD). The results suggested that elevated CRP levels might be a transdiagnostic biomarker in different psychiatric disorders, although multiple confounders might explain the presence of elevated CRP levels in a substantial portion of psychiatric patients [47]. Huang and Lin (2007) [59] reported higher hs-CRP levels in both MDD (*n* = 23) and BD-I (*n* = 13) groups compared to healthy controls (*n* = 31). No significant associations were found between CRP levels and MDD, even after adjusting for covariates; while a significant association was found between CRP levels and BD-I, even after adjusting for covariates (*p* = 0.043). A cross-sectional study aimed at investigating differences between CRP levels in patients with acute schizophrenia (*n* = 458), unipolar depression (*n* = 319), BD (*n* = 146), BD depression (*n* = 114) and BD mania (*n* = 32), did not find significant differences between groups [74]. However, more than one-third (38.6%) of the unipolar depression patients displayed high CRP levels (i.e., >3 mg/L) [74]. A cross-sectional study recruiting 1535 adolescents aged 13–16 years from a province-wide youth survey, did not find any association between CRP levels and depressive symptoms [49]. Chang et al. (2012) [50] evaluated whether CRP levels were associated with depressive symptoms and cognitive impairment in MDD patients. The authors found that among 149 recruited subjects, there was no association between CRP levels and HAM-D scores in the medication-free MDD subgroup.

A large cross-sectional study conducted by Menezes et al., 2017, including 14,821 Brazilian participants of the Brazilian Longitudinal Study of Adult Health (ELSA-Brasil), investigated the possible association between CRP levels and depression [68]. Current depression was assessed by Clinical Interview Schedule-Revised (CIS-R) and participants were divided into three groups: (a) negative at CIS-R and not using antidepressant; (b) negative at CIS-R but using antidepressants; (c) positive at CIS-R. No association between serum CRP levels, current depression, and use of antidepressants was found [68]. Another cross-sectional study, including 9459 Chinese middle-aged and elderly participants of the China Health and Retirement Longitudinal Study, did not find any correlation between CRP levels and depressive symptoms after adjusting for confounders [76]. A recent large cross-sectional study assessing serum hs-CRP levels and depression symptoms using CESD, in 26,638 healthy adults, recruited at the Cooper Clinic in Dallas, Texas, reported an association between hs-CRP levels and depression which could be more likely explained by obesity status [62]. An observational study investigated the association between CRP levels and depression also considering shared genetic and environmental factors [75]. Data were collected from 2577 twins and 899 singletons in Colombo, Sri Lanka. Depression was assessed using the revised Beck Depression Inventory (BDI-II) and the heritability of CRP levels was evaluated through Structural Equation Modelling. No association between CRP levels and depression was found. Moreover, in males the variance in CRP levels was explained by shared environment and non-shared environment, while in females it was explained by genetic and non-shared environment [75].

#### 3.1.2. Case-Control Studies

A recent case-control study compared 84 depressed patients, by classifying them in two groups, i.e., those with inflammation (CRP levels ≥ 3 mg/L) and those without inflammation [80]. The authors found, after adjusting for potential confounders, that the low-grade inflammatory group showed higher depression severity, somatic symptoms, state anxiety, and fatigue, but not anhedonia, compared to the group with inflammation. They also reported a poorer quality of life and an increase in guilty feelings, pessimism, concentration difficulties, and indecision [80].

A study recruiting 26,894 participants with a lifetime diagnosis of MDD, evaluated using the Composite International Diagnostic Interview (CIDI), were compared with 59,000 healthy control subjects regarding CRP levels [83]. CRP levels were significantly higher in patients with depression than in the control group (2.4 mg/L compared with 2.1 mg/L, *p* < 0.001). Furthermore, MDD patients showed more frequently a low-grade inflammation state (i.e., CRP > 3 mg/L) than control subjects (21.1% compared with 16.8%, respectively) [83].

A case-control study comparing 25 patients aged ≥55 years at their first depressive episode, assessed through Hamilton Depression Rating Scale (HAMD), and 27 age-matched healthy controls, found that subjects with current depressive disorders showed 40-fold higher CRP levels compared to control group [81]. The authors suggested that the late-onset depression was associated with higher CRP levels. Moreover, CRP levels were strongly and positively associated with depression severity [81]

Another study proposed CRP as a biomarker to differentiate MDD and BD type 2 (BD-II), by recruiting 88 BD-II, 72 drug-naïve MDD and 96 healthy controls [79]. The authors found that a baseline CRP level of 621.6 ng/mL could discriminate between MDD and BD-II in both depressed and euthymic states [79].

A case control study investigating the differences in serum CRP levels between elderly (≥60 years) depressed patients and healthy elderly individuals (202 with unipolar depression and 202 healthy subjects), found no significant differences between CRP levels in the two groups [86]. In addition, the authors reported that CRP levels were not associated with age and about 30% of the sample showed CRP level > 3 mg/L [86].

#### 3.1.3. Cohort Studies

A prospective cohort study investigated the relationship between long-term patterns of systemic inflammation and late-life depression symptomatology using the Atherosclerosis Risk in Communities (ARIC) Study, a large community-based prospective cohort study [94]. The study measured CRP levels and depressive symptoms (by using CESD) in 4476 participants over a 21-year period spanning from middle-to-late-life, at three moments (at the current moment, 14 years before, and 21 years before the current visit). Individuals with stable elevated CRP levels (>3 mg/L at all three visits) showed greater depressive symptomatology at older ages, after adjusting for covariates. Moreover, stable elevated CRP levels were associated with an increased risk for late-life depressive symptomatology [94].

A UK general population-based birth cohort study, named the Avon Longitudinal Study of Parents and Children (ALSPAC), evaluating CRP levels in 1561 participants, identified population sub-groups of young people characterized by different longitudinal patterns of CRP levels. Subjects who displayed a pattern of increasing CRP levels from childhood to early adulthood had a higher risk of moderate/severe depression at 18 years, compared to those who had persistently low CRP levels [29]. Subjects who displayed persistently elevated CRP levels also had increased ORs of moderate/severe depression at 18 years, even though this association was not statistically significant. Overall, an increase in low-grade inflammatory levels from childhood to early adulthood is strongly associated with the risk of developing depression in early adulthood [29].

A cohort study selected 1508 young individuals who were evaluated regarding the incidence of low-grade inflammation (i.e., CRP < 3 mg/L at baseline) and the depressive state using the Beck Depression Inventory (BDI) [100]. The authors found that the incidence of low-grade inflammation was more frequent in patients with depressive symptoms than among healthy controls (OR = 2.05; 95% CI = 1.31–3.21, *p* < 0.001). After adjusting for age, sex, metabolic syndrome, BMI, levels of physical activity, smoking, presence of hepatic steatosis, and waist circumference, it was confirmed the association of depressive symptoms and low-grade inflammation. New cases of inflammation were associated with depressive symptoms (OR = 1.76; 95% CI = 1.03–3.02, *p* = 0.04) [100].

Findings coming from the Great Smoky Mountains Study, a prospective cohort study investigating CRP levels in a cohort of 1334 adolescents and young adults, did not find any association between CRP levels and later depression status [89]. Multiple depressive episodes appeared to predict later CRP levels [89].

In 3397 older adults, an examination based on the English Longitudinal Study of Ageing studied the directional associations between CRP levels and depressive symptomatology [88]. Baseline high CRP levels were associated with depression symptoms severity. Baseline elevated depressive symptomatology was not associated with subsequent high CRP levels [88].

### 3.2. Studies on Gender Differences of CRP Levels in Depression

#### 3.2.1. Cross-Sectional Studies

A cross-sectional study assessed 6005 Finns aged >30 years for depression, using BDI and CIDI, and for CRP levels [54]. Higher BDI-21 scores were associated with higher CRP levels only in men (*p* < 0.001), even after adjustment for confounders [54]. Moreover, in men the more recent dysthymic disorder or at least moderate depressive episode was associated with higher CRP levels, compared to females (*p* = 0.006) [54]. Another cross-sectional study, based on the KNHANES, aimed at investigating the sex difference in the association between hs-CRP levels and depression, recruited 5483 Korean adults (2373 men and 3110 women) assessed with PHQ-9 (cut-off for depression > 10) [64]. Men with high hs-CRP levels (>3.0 mg/L) reported a higher prevalence of depression than those with lower levels (*p* < 0.0001). No statistically significant association was observed between hs-CRP levels and depression among women [64]. Similar findings were found in another Korean cross-sectional study, in which 596 participants (224 men and 345 women) from the Korean Social life, Health and Aging Project Health Examination (KSHAP-HE) cohort, were assessed for depressive symptoms through the CES-D and for serum CRP levels [71]. The authors reported that elevated CRP levels were independently associated with depressive symptoms in elderly Koreans, but this association was observed only in men both before and after adjusting for covariates [71].

Ford and Erlinger (2004) [55] found in 6914 participants (3154 men and 3760 women), recruited from the NHANES survey, that a history of MDD was associated with elevated CRP levels and that this association was much stronger among men than women. Moreover, CRP levels were higher among men who had a recent episode of depression and who had recurrent depression. A cross-sectional study evaluated whether there was a gender difference in the association between depressive episodes and hs-CRP [66]. From the genetically homogeneous Northern Finland 1966 Birth Cohort, 5269 participants (2641 male and 2828 female) were assessed with the Hopkins Symptom Checklist-25 (HSCL-25) and were measured CRP levels, by reporting that elevated CRP levels in males might increase the probability for severe current and recurrent depressive episodes. This association was not found in women [66]. Another cross-sectional study, including 764 (336 male and 579 female) individuals assessed through the Short Zung Self Rating Depression Scale (SZRDS), showed a positive correlation between hs-CRP levels and depression mood, exclusively in men [67].

A population-based study investigating the role of gender in the association between CRP levels and depressive severity, recruited 231 individuals (142 female and 89 men) with MDD from the Genome-Based Therapeutics Drugs Depression (GENDEP) study [63]. The subjects were assessed for hs-CRP levels and depression using the Montgomery Åsberg Depression Rating Scale (MADRS). Findings showed that higher CRP levels were significantly associated with greater overall depressive symptoms severity, which was significant among women but not in men. Moreover, women with higher CRP levels had an increase in specific depressive symptoms severity such as observed mood, cognitive symptoms, interest-activity, and suicidality [63]. A cross-sectional study included 563 adolescent girls, aged 12–18 years, aimed at investigating the association between hs-CRP levels and depressive symptomatology assessed through BDI-II [72]. Serum hs-CRP levels were 0.61 (0.30–0.88) mg/L in the non-depressed group, 0.97 (0.50–1.82) mg/L in the group with a mild depression score, 1.04 (0.57–1.60) mg/L in those with a moderate depression score, and 0.84 (0.45–2.64) mg/L in girls with severe depression (*p* < 0.001). Multinomial logistic regression analysis, controlling for covariates, showed that depression scores were positively associated with serum hs-CRP levels (*p* < 0.001) [72].

#### 3.2.2. Cohort Studies

A longitudinal study aimed at examining the association between depression and hs-CRP levels, found a positive association between BDI scores and serum hs-CRP levels only in women [90].

A 2010 longitudinal study investigated the association between depressive symptoms and CRP levels in 3302 mid-life women [92]. The assessment included CESD and blood CRP levels at the baseline and after 7 years. Results showed that higher CESD scores predicted higher CRP levels and vice versa over a 7-year period [92].

A retrospective cohort study evaluating the association between CRP levels and increased risk of *de novo* MDD, recruiting 1494 women randomly selected and assessed through the Structured Clinical Interview for DS-IV-TR Research Version, Non-Patient edition (SCID-I/NP), reported a hazard ratio for depression increased by 44% for each standard deviation increase in log-transformed hs-CRP, indicating that serum hs-CRP was an independent risk factor for de novo MDD in women [93].

#### 3.2.3. RCT Studies

A recent RCT evaluated the sex differences in the association between CRP levels and the response to antidepressant treatments [97]. Participants were assessed with HAMD-17 at baseline and at weeks 1, 2, 3, 4, 6, and 8 after the treatment. Elevated baseline CRP levels were associated with the worst antidepressant treatment outcome only in female samples [97].

### 3.3. Studies on Ethnic Differences of CRP Levels in Depression

#### Cross-Sectional Studies

A cross-sectional study evaluated the association between social integration, race/ethnicity, inflammation, and depressive symptoms in US participants through the NHANES survey [51]. Participants were administered PHQ-8 and measured hs-CRP levels. Social integration and CRP levels were found to operate independently in their association with depressive symptoms only for the white population but not the black or the Hispanic one [51]. A cross-sectional study evaluated whether specific symptom clusters were associated with CRP levels and whether race/ethnicity affected this association in a sample of 10,149 U.S. individuals from the NHANES survey [48]. Depressive symptomatology was assessed by PHQ-9, and serum hs-CRP was quantified. Somatic symptoms were related to CRP levels (*p* < 0.001), only in non-Hispanic white individuals [48].

### 3.4. Studies on Severity/Specific Cluster Domains in the Association between CRP Levels and Depression

#### 3.4.1. Cross-Sectional Studies

A study involving 5909 patients aimed at investigating the possible association between inflammation and specific depressive symptoms, found that CRP levels were associated with symptoms of fatigue (*p* < 0.001), restless sleep (*p* = 0.03), low energy (*p* = 0.02) and feeling depressed (*p* = 0.04) [73]. These associations were absent in patients under antidepressant medication [73].

Case and Stewart [48] evaluated if specific symptom clusters were associated with CRP levels, by recruiting a sample of 10,149 U.S. individuals from the NHANES survey. Depressive symptoms were assessed by PHQ-9, and serum hs-CRP was quantified. Somatic symptoms were related to CRP levels (*p* < 0.001) [48]. A recent network analysis investigated the association between inflammation and a specific depression phenotype [69]. PHQ-9 was administered to a sample of 4157 adults from the NHANES while hs-CRP levels were measured to identify possible inflammatory phenotypes of depression [69]. The elevated CRP group (>3 mg/L) showed greater symptom connectivity, concentration, psychomotor difficulties, and treatment-resistant depression [69].

A cross-sectional study evaluated the association between CRP levels, depressive symptoms, and cognitive impairment in 149 MDD patients treated with antidepressants for six weeks [50]. Scale for Depression (HAM-D), Continuous Performance Test (CPT), Finger-Tapping Test (FTT), and Wisconsin Card-Sorting Test (WCST) were administered to the sample. Baseline CRP levels were negatively and significantly associated with performance in the FTT and WCST after six weeks of treatment (respectively, *p* = 0.006 and *p* = 0.021), reporting a significant association between CRP levels and the domains of attention and executive cognitive function. After six weeks of treatment, patients with higher baseline CRP levels still exhibited poor psychomotor speed and poor executive functioning [50].

A cross-sectional study recruiting 43,896 adults examined the association between CRP levels and depression in terms of executive functioning [56]. MINI and Ruff Figural Fluency Test (RFFT) were administered to the sample. The study reported that depression and higher CRP levels were both associated with worse executive functioning, even after covariates adjustment. Moreover, depressed subjects with higher levels of CRP showed poorer executive functioning than the control individuals [56].

#### 3.4.2. Cohort Studies

A prospective cohort study investigated whether depression and apathy in the elderly subjects could be associated with CRP levels [91]. The study was conducted on 599 subjects assessed annually from age 85 to 90 through Mini-Mental State Examination (MMSE) and the Geriatric Depression Scale 15-items (GDS-15). Higher baseline CRP levels were associated with severe depressive symptoms but not apathy [91].

### 3.5. Studies on Genetic Correlation and Single-Nucleotide Polymorphisms (SNPs) in the Association between CRP Levels and Depression

#### 3.5.1. Cross-Sectional Studies

A cross-sectional study explored whether CRP levels may covary with depressive symptoms due to allelic variation in the CRP gene [57]. The study recruited 868 healthy community volunteers who were assessed through CESD, plasma CRP levels and genome CRP SNPs. No direct association between CESD and CRP levels was found. One haplotype (T-G-C) was associated with CRP level (*p* = 0.014), but no one was related to depressive symptoms. Plasma CRP levels were predicted by the interaction of the A-G-T haplotype with depressive symptomatology (*p* = 0.009). Higher CESD scores were associated positively with CRP levels among individuals with the A-G-T haplotype (*p* = 0.004) [57].

A cross-sectional study exploring whether CRP SNPs were related to depressive symptoms and antidepressants efficacy assessed 440 patients with first-episode depression through HAMD-17, finding gender-specific SNP differences [65]. In particular, male patients with SNP rs1800947G exhibited lower insomnia scores while rs2794521CC showed lower scores of anxiety/physical symptoms, total HAMD-17 score; female patients with rs2794521TT had higher scores of insomnia and lower antidepressants efficacy [65]. A large cross-sectional study included 3700 men ≥ 70 years in determining if polymorphisms of SNPs rs1130864 and rs1205 were associated with MDD [45]. GDS-15 and genome CRP SNPs evaluations were used. The odds of depression increased by 2% (95% CI = 1–4%) for every unit (mg/L) increase of CRP and nearly doubled for men with CRP ≥ 3 mg/L vs. < 1 mg/L. Nevertheless, the association between high CRP (i.e., ≥3 mg/L) and depression was no longer significant after the analyses were adjusted for confounders. Men with the CT and TT genotypes of rs1130864 had greater odds of CRP ≥ 3 mg/L than CC carriers, but there was no association between this polymorphism and the presence of prevalent depression. The G > A polymorphism of SNP rs1205 was associated with a 24% lower CRP concentration than other genotypes. Men with the rs1205 AA genotype had greater odds of having clinically significant depression than participants with the GA and GG genotypes [45]. Similarly, a cross-sectional study studied the association between variants in the CRP gene that influence protein levels and depression in 990 people aged ≥65 years whose psychopathology was assessed through MINI and CESD [46]. The minor alleles of rs1130864 and rs1417938 were associated with a decreased risk of depression in women (*p* = 0.002). Conversely, rs1205 was found to be nominally associated with both an increased risk of depression and lower circulating CRP levels in women [46].

#### 3.5.2. Case-Control Studies

A case-control study investigated whether inherited CRP allelic variations could covary depressive symptoms, in 200 patients, aged 18–65, who were assessed for CRP blood levels and genome CRP SNPs [87]. Patients with a positive family depression history had higher CRP blood levels. Specific inherited CRP SNPs (A allele in rs1417938 and C allele in rs1205) may be responsible for up-regulating serum CRP levels and thus bated with depression occurrence [87]. Another case-control study investigated whether CRP SNPs could regulate plasma CRP levels if inherited CRP allelic variations may covary with depressive symptoms [85]. CRP blood levels and genome CRP SNPs were evaluated in 60 MDD patients with family depression history and 60 healthy control volunteers. A significantly higher circulating CRP level was found in the first group [85].

### 3.6. Studies Investigating the Association between CRP Levels and Antidepressant Treatment

#### 3.6.1. Cross-Sectional Studies

A large cross-sectional study investigated the association of CRP levels and psychological distress mediated by antidepressants in 10,363 UK adults, found robust associations of log-CRP and General Health Questionnaire (GHQ) among antidepressant users but not for non-users in both cross-sectional (*p* = 0.01 vs. 0.06, *p* = 0.28) and longitudinal models (*p* = 0.006 vs. 0.04, *p* = 0.39 two waves post-baseline) [60].

A cross-sectional study investigating the effects of agomelatine on CRP levels in 30 MDD patients assessed with MINI, HAMD, and the Snaith-Hamilton Pleasure Scale (SHAPS), proved that agomelatine significantly reduced depressive symptoms and CRP levels [53]. Higher CRP level variation was associated with higher baseline HAMD scores at the baseline [53].

An observational study analyzed the association between CRP levels with a worse response to escitalopram and a better response to nortriptyline in consideration of genetic disposition to inflammation [77]. A higher polygenic risk score for CRP was found to be associated with a slightly better response to escitalopram and a slightly worse response to nortriptyline, reflected in a statistically significant interaction between polygenic risk score and drug (*p* = 0.0093) [77].

We already mentioned a cross-sectional study evaluating the correlation of CRP with depressive symptoms and cognitive impairment in 149 MDD patients treated with fluoxetine or venlafaxine for six weeks [50]. Baseline levels of CRP were not correlated with baseline HAM-D scores (*p* = 0.606) but were significantly associated with treatment response at week 2 (*p* = 0.020) when patients with higher CRP levels had a poorer treatment response. CRP levels increased significantly after six weeks of treatment (*p* < 0.001), and CRP levels remained significantly high in patients with higher baseline levels (*p* < 0.001) [50].

#### 3.6.2. Case-Control Studies

A case-control study evaluated the association between MDD clinical features and hs-CRP levels in a sample of 103 TRD patients and 103 non-TRD patients [84]. Depressive symptoms were investigated through HAMD-17. In the TRD group, the disease course was longer, the onset was earlier, and the educational level was lower. The HAMD score (*p* = 0.031), anxiety/somatization factor score (*p* = 0.015), and sleep disorder (*p* = 0.029) of TRD patients were positively associated with hs-CRP level, while the onset age (*p* = 0.009) was negatively correlated with the hs-CRP level [84].

A case-control study explored CRP levels in MDD subjects and its phenotypic associations in 102 TRD patients who currently experience depression, 48 treatment-responsive patients not undergoing depression, 48 patients not receiving medication, and 54 healthy volunteers [78]. Higher CRP levels were found in MDD patients compared to controls, and higher CRP levels were found in TRD patients compared to treatment-responsive MDD [78].

A case-control study examining CRP levels to evaluate the impact of SSRI treatment in 32 MDD individuals compared to 20 healthy subjects, following measuring CRP levels before and after SSRI treatment, reported a significant reduction after the treatment, regardless of symptom reduction [82].

#### 3.6.3. RCT Studies

A retrospective study including 75 adult inpatients with MDD aimed at investigating the possibility of using CRP levels as predictors of antidepressant treatment response, found that subjects with high CRP levels had higher HDRS-17 scores, showed lower responses after 3 and 4 weeks of treatment, and lower remission rates [95].

The previously mentioned RCT evaluating the sex differences in the association between CRP levels and the response to antidepressant treatments, found that higher baseline CRP levels were found to be associated with a lower baseline-to-week-8 HAMD-17 reduction in females (*p* < 0.0001) but not in males (*p* = 0.632) [97].

A multicenter open-label RCT tested the hypothesis that CRP levels can predict differential response to escitalopram and nortriptyline [98]. CRP levels at baseline differentially predicted treatment outcomes with the two antidepressants. For patients with low levels of CRP (i.e., <1 mg/L), the improvement at MADRS total score was 3 points higher with escitalopram. For patients with higher CRP levels, the improvement in the MADRS score was 3 points higher with nortriptyline [98]. A RCT investigated the efficacy of anti-inflammatory (celecoxib) augmentation of antidepressant treatment in MDD patients and evaluated whether treatment response depended on baseline inflammation levels [96]. Data from 119 participants showed no evidence of superior efficacy of celecoxib augmentation over placebo and neither that pretreatment inflammation levels could modify the effect of celecoxib augmentation versus placebo [96].

### 3.7. Quality Assessment

Based on our judgment, 40 studies were rated as “good” studies [16,45,46,48,49,51,52,53,54,55,56,57,58,59,60,61,62,63,64,65,66,68,71,72,73,75,76,78,81,83,88,89,90,92,93,94,96,97,98,100] and 16 as “satisfactory” studies [47,50,67,69,70,74,77,79,80,82,84,85,86,87,91,95]. The main misses were the lack of justified and satisfactory sample size calculation before the study, the absence of non-respondent information and, in some studies, the lack of comparability (Supplementary: Newcastle-ottawa quality assessment scale Tables S1–S4).

## 4. Discussion

Overall, an increasing amount of evidence has suggested the presence of a dysregulation in the inflammation system in depressed patients [13,15,16,101]. Different pathways seem to be involved such as the kynurenine pathways hypothesis of depression in which there is excessive activation of the indoleamine-2,3-dioxygenase (IDO) [102]. This enzyme is present in microglia, astrocytes, and neurons and catabolizes tryptophan into kynurenine, a neurotoxic substrate, and it is responsible of reducing the amount of available tryptophan to produce serotonin [103]. Moreover, enzymes of inflammation such as manganese superoxide dismutase (MnSOD), myeloperoxidase (MPO), and inducible nitric oxide synthase (iNOS) were involved in the genesis of depressive disorder, by actively inducing the production of free radical, fatty acid, cellular DNA, and other factors that may lead to brain damage [104,105]. Indeed, several studies demonstrated an association between recurrent depressive disorders and increased activity of the enzymes mentioned above [105,106,107,108]. Another mechanism which was suggested to be implicated is represented by the oxidative stress which may predispose to an increased activity of reactive oxygen species (ROS) [10]. An imbalance between the antioxidant system and oxidizing agents may lead to macromolecules damage, alteration of normal cell signaling pathways, and structural and functional alteration [109,110,111]. Specific regions of the hippocampus (CA1 and CA4 region), cells in the dorsolateral region of the striatum, and neurons in the third and fifth layer of the cerebral cortex are most sensitive to damage [110]. Moreover, in patients suffering from depression, an increased expression of malondialdehyde, NO, and thiol protein group was reported. This is associated with reduced total antioxidant status, which can lead to deteriorated efficiency of operational memory, declarative memory, and verbal fluency [112,113]. Furthermore, oxidative and inflammatory pathways are strictly interconnected [114]. Indeed, oxidative stress induces inflammation through Nuclear Factor-kB (NF-kB) and consequently may cause an increased production of free radicals [114].

Furthermore, researchers also investigated the role of cytokines and acute-phase proteins in order to evaluate if it is possible to recognize specific inflammatory biomarkers for depression [16,115,116,117]. In particular, a research field of interest consists of identifying specific subgroups of MDD patients with a specific inflammatory pattern which may be associated with a different treatment response and, hence, needed a personalized, tailored treatment strategy. Among these inflammatory biomarkers, numerous studies focused on depression and CRP [15,29,56,115,118]. In fact, CRP is an acute-phase reactant protein, produced by the liver in response to inflammatory state and it is easily and cheap to be measured through a blood sample [24,30].

Overall, the present systematic review reported that most studies here retrieved, found a positive association between elevated CRP levels and depression [29,51,52,54,55,61,63,64,66,67,69,70,71,72,73,80,81,83,90,91,92,93,94,100]. Conversely, other studies did not confirm this association [49,50,62,68,74,76,86,88,89].

Specifically, CRP levels seem to be associated with depressive symptoms severity [29,63,72,80,81,90,91,92]. Furthermore, several studies investigated whether there was a specific pattern of depressive symptoms related to higher CRP levels, suggesting an association with symptoms of fatigue, restless sleep, low energy, concentration difficulties, poor psychomotor speed, and poor executive functioning [50,56,63,69,73]. In addition, CRP levels seem to be related to somatic symptoms [48] and not to apathy [91].

Higher CRP levels were observed in atypical MDD with respect to the typical manifestation [58], and were associated with reduced quality of life [80] and late-onset depression [81]. Moreover, hs-CRP levels seem to be an independent risk factor of depression and elevated hs-CRP levels could be a predictor of the onset of MDD [93]. Finally, stable elevated CRP levels were associated with increased risk for late-life depression symptoms [80] and the occurrence of multiple depressive episodes seems to improve later CRP levels, maybe by increasing the risk for cardiovascular and metabolic disease [89].

Evidence suggested also that CRP levels may vary among gender and/or ethnic differences. Lee et al. (2019) [64] reported a higher prevalence of depression in men with high CRP levels. The association was not observed in women. These results are consistent with other studies in which a positive association between CRP levels and depression was observed only in males. On the other hand, other studies displayed opposite results with a positive association between CRP levels and depression only among women [63,72,90,92,97]. Few studies reported that CRP levels were independently associated with depressive symptoms only in the white non-Hispanic population [48,51]. These results indicate a biological difference, yet to be understood, between gender/race/ethnicity that can independently modify the relationship between CRP levels and depression.

Among the studies included in the present systematic review, only three studies investigated CRP as a biomarker to differentiate psychiatric disorders [59,74,79]. Chang et al. (2017) [79] suggested that CRP levels of 621.6 mg/L could discriminate between MDD and BD-II in both depressed and euthymic states. The same results come from a study by Hanug and Lin (2007) [59] that reported persisted high CRP levels in BD-I after adjusting for covariates and not in MDD. On the contrary, in another study no differences were observed in CRP levels across different psychiatric diagnoses [86].

Furthermore, it would appear that there is a subgroup of depressed patients who showed a low-grade inflammatory state (i.e., CRP > 3.0 mg/L) [29,47,61,69,74,80,83,86,100], corroborating the hypothesis that inflammation might contribute to developing some types of depression, but not all of them [75]. Indeed, about a third of all depressed patients seem to express CRP levels > 3.0 mg/L [86,119,120]. Data were confirmed by a recent meta-analysis that included 13,541 depressed patients and 155,728 controls [121]. In 27% of the depressive patients there was a low-grade of inflammation (i.e., CRP > 3.0 mg/L) and over half of the patients showed a mildly elevated CRP level [121]. This may point out that a chronic low-grade inflammation could be associated with a different MDD subgroup with a distinct etiopathogenesis, clinical course, treatment response, and prognosis [13,72,121]. Indeed, 30% of patients with depression do not get relief from standard antidepressant therapy and this may be due to this low-grade inflammatory state [122].

Based on the abovementioned inflammatory theory of depression, several studies investigated the association between CRP levels and antidepressant treatment [123]. A wide range of studies reported that elevated levels of CRP were associated with TRD patients [50,60,78,84,95,97]. Conversely, other studies showed that lower levels of CRP were associated with a better and faster response to SSRI treatment [63,98]. Indeed, anti-inflammatory action of SSRI and serotonin and noradrenaline inhibitors (SNRIs) has been hypothesized through which they can indirectly reduce depressive symptoms [124,125,126], even though the findings are still contradictory [77]. Little evidence reported that antidepressant treatment may decrease CRP levels [82,124], although a recent meta-analysis conducted by Wiedlocha et al. (2018) [127] did not show significant effect on CRP levels using antidepressants. Based on the inflammatory theory, anti-inflammatory drugs (e.g., non-steroidal anti-inflammatory drugs or anti-cytokine) could be useful in the treatment of depression [128,129,130,131], despite findings so far are contradictory [96,132,133].

Different mechanisms were assumed to explain how a low-grade inflammation may interact with the severity of depressive symptoms, including cognitive impairment, and with treatment resistance in MDD patients [56,121,134]. A possible explanation is that low-grade inflammation (i.e., CRP > 3 mg/L) may lead to cerebral inflammation by decreasing neurotrophic support, oxidative stress damage, increasing glutamatergic excitotoxicity, and affecting neuronal serotonin transporter activity [135] which, then, may determine a microstructural disintegration which predominantly affects frontal pathways and corresponding executive function [136]. Moreover, it can also affect the dopaminergic neurons related to cognitive function, including psychomotor speed, memory, and executive cognitive function [137,138,139,140]. For these reasons, interventions that reduce inflammation may improve cognitive functioning in depression [56]. Moreover, patients with MDD might be stratified for CRP levels to distinguish different clinical profiles that could be responsive to second-line treatment with anti-inflammatory drugs [78].

Furthermore, several studies have investigated the role of SNPs in the association between CRP levels and depression with mixed results [45,46,57,65,85,87].

However, despite the encouraging and interesting findings coming from the present systematic review, there are several limitations in the present review to be discussed. Firstly, most studies display a methodological heterogeneity in terms of study design, different sample sizes, inclusion/exclusion criteria, various diagnostic tools, and recruitment settings. Secondly, not all included studies controlled for potential confounding variables in the association between depression and inflammation and, hence, this may limit the generalizability of the findings in those studies which report positive associations between CRP levels and depression. Thirdly, the socio-demographic characteristic of the samples is extremely dis-homogeneous in terms of gender, race, ethnicity, etc. In this regard, few studies specifically investigated (if any) differences occur in the association between CRP levels and depression, considering gender, ethnicity, type of MDD severity, clinical course, illness duration, type of concomitant treatment and so forth. Furthermore, from a clinical point of view, studies here retrieved showed different phases of illness (i.e., acute, and chronic phase, remitted patients, early-onset or late-onset patients, etc.). Finally, antidepressant treatment has not been always reported (i.e., patients with stable antidepressant treatment, patients with not stable antipsychotic medication, free-drug patients, naïve-drug patients, not specific antidepressant medication, etc.).

Further studies are needed in order to better understand the core mechanism through which CRP may interact in depression and which role CRP may have in characterizing subgroups of depressed patients and guide treatment strategies. More methodologically homogeneous and more geographically defined studies also focusing on gender and ethnicity CRP variability could be useful in better understanding biological differences among different sexes and races and guide more personalized and patient-tailored interventions. Similarly, further studies should be implemented for investigating the different MDD phases and the different clinical depression subtypes, in order to identify whether a specific subgroup of MDD patients may benefit from monitoring CRP levels from a clinical and therapeutic point of view. Furthermore, there is the need for more studies that investigate how CRP and inflammation status may determine changes in the CNS of depressed patients, by implementing neuroimaging studies. Moreover, more RCT studies are needed in order to investigate the role of antidepressant and anti-inflammatory therapy in depressed patients with a low-grade of inflammation.

## 5. Conclusions

In conclusion, this systematic review provides robust evidence about the potential role of CRP and its blood levels in depression. Indeed, patients with elevated CRP levels seem to be associated with a greater symptom severity, specific pattern of depressive symptoms and a worst treatment response, although it is still unclear if inflammation may contribute directly to the pathogenesis of depression or whether it may rather be a consequence of the illness and covariates interaction. Moreover, about one-third of depressed patients showed a low-grade inflammatory state (i.e., CRP > 3.0 mg/L), by suggesting the presence of a different MDD subgroup with a distinct etiopathogenesis, clinical course, treatment response, and prognosis which may benefit of a monitoring of CRP levels and might potentially respond to anti-inflammatory treatments. These findings are indeed extremely relevant for the development of new interventional strategies and in order to better understand if CRP may be considered a useful biomarker for depression.

## Figures and Tables

**Table 1:** Summary of included studies.

Study	Study Design	Primary and Secondary Outcomes	Participants Characteristics	Assessment	Main Findings	Is There an Association between CRP Levels and Depression?
[45]	Cross-sectional study (Australia)	To determine if polymorphisms of SNPs rs1130864 and rs1205 are associated with prevalent depression	3700 men aged > or = 70 years	GDS-15 PCS Genome CRP SNPs	The odds of depression increased by 2% (95% CI = 1–4%) for every unit (mg/L) increase of CRP and nearly doubled for men with CRP > or = 3 mg/L vs < 1 mg/L (OR = 1.95, 95% CI = 1.27–2.98). Nevertheless, the association between high CRP (> or = 3 mg/L) and depression was no longer significant after the analyses were adjusted for smoking, age, body mass index (BMI), and PCS Men with the CT and TT genotypes of rs1130864 had 1.36 (95% CI = 1.13–1.63) and 2.31 (95%CI = 1.65–3.24) greater odds of CRP > or = 3 mg/L than CC carriers, but there was no association between this polymorphism and the presence of prevalent depression The G > A polymorphism of SNP rs1205 was associated with a 24% (95% CI = 16–32%) lower CRP concentration than other genotypes. Men with the rs1205 AA genotype had 1.66 (95% CI = 1.07–2.57) and 1.67 (95% CI = 1.08–2.58) greater odds of having clinically significant depression than participants with the GA and GG genotypes, respectively	Depressive symptoms in later life are unlikely to be caused by an increase in the serum concentration of CRP Risk of depression was greater amongst people who carry the rs1205 G > A genetic polymorphism of the CRP gene CRP may be a compensatory response to external insults that predispose to depression
[46]	Cross-sectional study (France)	Association between variants in the CRP gene that influence protein levels and depression	990 people aged at least 65 years	CESD Diagnosis of current major depression based on the MINI and according to DSM-IV criteria Genome CRP SNPs	The minor alleles of rs1130864 and rs1417938 were associated with a decreased risk of depression in women (*p* = 0.002) rs1205 was found to be nominally associated with both an increased risk of depression and lower circulating CRP levels in women	Variants of the CRP gene thus influence circulating CRP levels and appear as independent susceptibility factors for late-life depression
[47]	Cross-sectional study (Italy)	To compare CRP (i.e., serum CRP > 3 and ≤10 mg/L) in patients with MDD, BD and OCD	388 inpatients, (156 MDD, 135 BD, 97 OCD)	Blood CRP levels	Considerable elevated CRP levels were found among the three groups (36.5%, 47.4%, and 29.9% in MDD, BD, and OCD, respectively) without any significant differences across groups In the whole sample, elevated CRP levels variations were only partially attributable to potential confounders All groups presented considerable rates of cardiovascular risk factors	CRP levels may be a transdiagnostic feature of different psychiatric disorders Other confounder mechanisms may explain the presence of elevated CRP levels in a substantial portion of psychiatric patients
[48]	Cross-sectional study (USA)	To evaluate whether specific symptoms clusters are strongly associated with CRP levels and if race/ethnicity may affect this association	10,149 adults who participated to the NHANES	PHQ-9 serum hs-CRP levels	Total (*p* < 0.001), somatic (*p* < 0.001), and nonsomatic (*p* = 0.001) depressive symptoms were positively associated with CRP levels in individual models; in the simultaneous model that included both symptom clusters, only somatic symptoms (*p* < 0.001) remained associated with serum CRP levels Among non-Hispanic Whites, the pattern of results was identical to the full sample; only somatic symptoms (*p* < 0.001) remained related to serum CRP levels in the simultaneous model	A positive association was found between CRP levels and depressive symptomatology cluster
[49]	Cross-sectional study (Canada)	To examine the association between hs-CRP concentrations and depressive symptoms in youth	1535 adolescents (aged 13–16)	PDS hs-CRP levels	No significant association was found between CRP levels and depressive symptomatology	No association between hs-CRP levels and depressive symptoms
[50]	Cross-sectional study (China)	To evaluate the association between CRP, depressive symptoms and cognitive impairment in MDD patients treated with antidepressants (venlafaxine and fluoxetine) for 6 weeks	149 MDD subjects (M = 42 F = 107)	DSM criteria HAM-D CPT FTT WCST plasma CRP levels	Baseline CRP levels were not associated with baseline HAM-D scores (*p* = 0.606) Baseline CRP levels were significantly associated with treatment response at week 2 (*p* = 0.020). Patients with higher CRP levels had a poorer treatment response Baseline CRP levels were negatively and significantly associated with performance in the FTT and WCST after 6 weeks of treatment (respectively, *p* = 0.006 and *p* = 0.021) CRP levels significantly increased after six week of treatment (*p* < 0.001) CRP levels remained significantly high in patients with higher baseline CRP levels (*p* < 0.001)	The cognitive function of MDD patients with high baseline CRP levels may remain impaired even after antidepressant treatment that improves depressive symptoms
[51]	Cross-sectional study (USA)	Association between social integration, race/ethnicity, inflammation, and depressive symptoms	5634 participants aged 40 and older from the NHANES	PHQ-8 hs-CRP levels	Social integration and CRP operate independently in their association with depressive symptoms CRP levels were associated with depressive symptoms for white population, but not in the black or Hispanic one	Possible differences in how CRP levels are associated with depressive symptoms based on race/ethnicity
[52]	Cross-sectional study (Republic of Korea)	Examine the association between serum hs-CRP levels and depressive symptoms in adults and explore the potential moderating effects of age, sex, BMI, and aerobic physical activity on the association between hsCRP levels and depression	10,702 Adults (≥19 years) (M = 4746; F = 5956)	PHQ-9 serum hs-CRP levels	hs-CRP levels were associated with depressive symptoms (OR = 1.41, 95% CI = 1.07–1.84) and with non-geriatric population (in M, OR = 1.92; 95% CI = 1.12–3.31; in F: OR = 1.56; 95% CI = 1.02–2.39) A significant increased OR for developing depressive symptoms was observed only in adults with hs-CRP levels > 3.0 mg/L (OR = 1.40; 95% CI = 1.06–1.85) In subgroup analyses, the association between hs-CRP levels and depressive symptomatology was observed only among obese adults (OR = 1.81; 95% CI = 1.03–3.18) and among participants without a regular aerobic physical activity (OR = 2.13; 95% CI = 1.19–3.81)	A significant positive association between CRP levels and depressive symptoms.
[53]	Cross-sectional study (Italy)	To investigate the effects of agomelatine on CRP levels in MDD patients and whether CRP variations are associated with clinical improvement	30 adult MDD outpatients (M = 12 F = 18)	Diagnosis of current MDD based on the MINI and according to DSM-IV criteria HAM-D SHAPS Serum CRP levels	Agomelatine was effective in the treatment of MDD, with a significant reduction in HAM-D, SHAPS scores, and CRP levels Remitters showed a significant difference in CRP levels after 12 weeks of agomelatine Higher CRP level variation was associated with higher baseline HAM-D scores	Agomelatine was associated with a reduction in circulating CRP levels in MDD patients More prominent CRP level variation was associated with more severe depressive symptoms at baseline
[54]	Cross sectional study (Finland)	To evaluate whether depression is independently associated with elevated CRP levels	6000 Finns aged > 30 years (M = 2784F = 3257)	BDI-21 CIDI serum hs-CRP levels	Higher CRP levels were associated with higher BDI-21 scores (𝛽 = 0.08; *p* < 0.001). The association persisted only in men, after adjustment for covariates Recent dysthymic disorder or at least moderate depressive episode were associated with higher CRP levels in men (𝛽 = 0.06; *p* = 0.006)	Inflammation may induce depressive symptoms in men
[55]	Cross-sectional study (USA)	To determine the association between MDD and elevated CRP levels in a nationally representative US cohort	6914 noninstitutionalized (M = 3154 F = 3760) aged 18–39 yy	DIS Blood CRP levels	A history of MDD was associated with elevated CRP level (OR = 1.64; 95% CI = 1.20–2.24) The association between depression and CRP levels was much stronger among men than among women Compared with men without a history of depression, CRP levels were higher among men who had a more recent (within 1 year) episode of depression (adjusted OR = 3; 95% CI = 1.39–6.48) and who had recurrent (> or = 2 episodes) depression (adjusted OR = 3.55; 95% CI = 1.55–8.14)	Positive association was found between CRP levels and depression in men
[56]	Cross-sectional study (USA)	To investigate whether the combination between CRP levels and depression is associated with worse executive functioning	43,896 adults aged 44.13 years	MINI RFFT plasma CRP/hs-CRP levels	Depression (*p* < 0.001) and higher log-transformed CRP levels (*p* < 0.001) were associated with worse executive functioning Patients with higher levels of log-CRP levels showed differentially poorer executive functioning (*p* < 0.001) regardless of age	Depressed patients with higher CRP levels were poorer in executive functioning, even in early adulthood
[57]	Cross-sectional study (USA)	To explore whether plasma CRP levels may covary with depressive symptomatology as a function of allelic variation in the CRP gene	868 healthy community volunteers	CESD Plasma CRP levels Genome CRP SNPs	No direct association CESD-CRP was found. One haplotype (T-G-C) was associated with CRP level (*p* = 0.014), but no one was related to depressive symptoms Plasma CRP was predicted by the interaction of A-G-T haplotype with depressive symptomatology (*p* = 0.009) Higher CESD scores were associated positively with CRP levels among individuals with the A-G-T haplotype (*p* = 0.004)	Haplotypic variation in the CRP locus moderates an association of depressive symptoms with circulating CRP
[58]	Cross-sectional study (US)	To evaluate the association between depression subtypes with inflammatory state	19 atypical MDD patients, 93 non-atypical MDD, 1682 without MDD	DSM-IV CIDI	Atypical MDD was related with higher CRP levels than those with non-atypical MDD (*p* = 0.03) or without MDD patients (*p* = 0.005)	A positive association was found between CRP levels and atypical MDD
[59]	Cross-sectional study (China)	To assess the difference in serum hs-CRP levels between BD-I and MDD	23 MDD13 BD-I (manic episodes)31 healthy controls	SCID serum hsCRP	Both MDD and BD-I subjects showed higher hs-CRP levels than the healthy control group After adjustment for confounders, no statistically significant association was observed between MDD and hs-CRP levels (*p* = 0.172) A statistically significant association was observed between BD-I and hs-CRP levels (*p* < 0.001)	No association was found between CRP levels and MDD
[60]	Cross-sectional study (UK)	Associations of CRP and psychological distress mediated by antidepressants, supporting an inflammatory depression subtype	10,363 UK adults aged 16–98	GHQ Plasma CRP levels	Robust associations of log-CRP and GHQ were seen for antidepressant users but not for non-users in both cross-sectional (coeff: 0.54, *p* = 0.01 vs. 0.06, *p* = 0.28) and longitudinal models (coeff: 0.57, *p* = 0.006 vs. 0.04, *p* = 0.39 two waves post-baseline) suggesting evidence for the existence of an inflammatory depression subtype	Inflammatory depression subtype may present a stronger association of depressive symptomatology with inflammation (blood CRP levels) among antidepressant users than non-users
[61]	Cross-sectional study (Republic of Korea)	To examine the association between hs-CRP levels and depression	5447 participants coming from KNHANES VII-1 study	PHQ-9 serum hs-CRP levels	High CRP levels (>3.0 mg/L) were associated with higher rate of depression than in those participants with low hs-CRP levels (21.5% vs. 19.8%; *p* = 0.007) Serum hs-CRP was independently associated with the PHQ-9 total score (B = 0.014, 95% CI = 0.008–0.020) Elevated hs-CRP levels were significantly associated with an increased risk of depression in younger adults (adjusted OR = 1.44; 95% CI = 1.01–2.07)	A significant positive correlation between high hs-CRP levels and depression in younger adults
[62]	Cross-sectional study (USA)	To determine the association between hs-CRP levels and depression in a large sample of healthy adults	26,638 healthy adults	serum hs-CRP levels 10-item CESD	Weak association between hs-CRP levels and depressive symptoms (OR = 1.06 per mg/L, 95% CI = 1.03–1.09 for F; OR = 1.05 per mg/L, 95% CI = 1.02–1.09 for M) which became insignificant when controlling for BMI in F (OR = 1.02 per mg/L, 95% CI = 0.98–1.05) and M (OR = 1.02 per mg/L, 95% CI = 0.98–1.05) Adjusting for antidepressant and statin use did not affect the association between hs-CRP levels and depressive symptoms in F (OR = 0.99 per mg/L, 95% CI = 0.96–1.03) or M (OR = 1.01 per mg/L, 95% CI = 0.97–1.05) Levels of hs-CRP levels were not associated with depression independent of BMI in a predominantly white, male population of higher socioeconomic status	Associations between hs-CRP levels and depression may be explained by obesity
[63]	Cross-sectional study (Germany)	To evaluate the association between CRP levels and depression severity, including specific depressive symptoms	231 MDD patients (F = 142 M = 89) recruited from GENDEP study	MADRS HAM-D BDI serum CRP levels	Higher CRP levels were significantly associated with higher scores at MADRS (*p* = 0.02), which was significant among women (*p* = 0.02) but not in men (*p* = 0.68) In women, CRP levels were associated with specific symptoms: observed mood (*p* = 0.003), cognitive symptoms (*p* = 0.01), interest-activity (*p* = 0.02) and suicidality (*p* = 0.05)	Low-grade of inflammation may be associated with a subtype of depression and gender differences
[64]	Cross-sectional study	To evaluate the sex difference in the relationship between CRP and depression	5483 Korean adults (2373 men and 3110 women) recruited from KNHANES	PHQ-9 serum hs-CRP	Men with high hs-CRP levels displayed higher prevalence of depression than those with low hs-CRP levels (8.90% vs. 3.65%; *p* ≤ 0.0001) No statistical significant association between CRP and depression was observed among women	Positive correlation between CRP and depression among man but not in women
[65]	Cross-sectional study (China)	To explore whether CRP SNPs are related to depressive symptoms and antidepressants efficacy	440 patients with first-episode depression	Genome CRP SNPs HAMD-17	Male patients with SNP rs1800947G exhibited lower insomnia scores and rs2794521CC exhibited lower scores of anxiety/physical symptoms, total HAMD17 score Female patients with rs2794521TT exhibited higher scores of insomnia and lower antidepressants efficacy	Some SNPs are associated with depressive symptoms in a specific way based on gender Some SNPs may be a predictor of the efficacy of antidepressants in female patients
[66]	Cross-sectional study (Finland)	To investigate whether depressive episodes are associated in both genders with hs-CRP levels	5269 participants (M = 2641 F = 2828)	Blood CRP levels HSCL-25	In male subjects, elevated hs-CRP levels (> or = 1.0 mg/L) increased the probability for severe current and recurrent depressive episodes 1.7-fold and 3.1-fold, respectively hs-CRP levels > 3.0 mg/L increased the probability for recurrent depression up to 4.1-fold	A positive association was found between CRP levels and severe depression in men
[67]	Cross-sectional study (Finland)	Association between hs-CRP levels and depressed mood among the elderly	764 subjects aged 70 years or older	Serum hs-CRP levels SZSRDS	High hs-CRP levels predict a higher incident of higher SZSRDS score and depressed mood among older men	A positive association was found between hs-CRP levels and depressed mood only in men
[68]	Cross-sectional study (Brazil)	To investigate relationship between serum CRP levels and depression	14,821 participants recruited from ELSA-Brazil study	CIS-R serum CRP levels	Neither current depression, nor antidepressant use was statistically associated with elevated CRP levels	No association between CRP levels and depression
[69]	Cross-sectional study (USA)	To investigate the possible association between inflammation and a specific phenotype of depression	4157 participants from NHANES (F = 51.3%) with mean age of 47.59	PHQ-9 hs-CRP levels	CRP group had greater symptom connectivity and concentrating/psychomotor difficulties Several symptom-symptom association were moderated by CRP levels	CRP levels can identify a particular depression phenotype with specific symptoms and treatment response
[70]	Cross-sectional study (Czech Republic)	To confirm the possible association between depression and CRP levels	6126 individuals (45–69 yy) (M = 2829; F = 3297)	CESD serum hs-CRP levels	CESD score 16+ in M: 13% CESD score 16+ in F: 22.8% Strong association between depressive symptoms and CRP in both genders (0.57 (0.10–1.04) mg/L in M and 0.61 (0.27–0.96) mg/L in F) Mean CRP concentrations of 0.60 mg/L (95% CI = 0.32–0.87) higher in subjects with depressive symptoms compared to those without depressive symptoms. Linear association between CRP concentration and CESD scale with an increase in CRP of 0.23 mg/L (95% CI = 0.12–0.34) in age-sex adjusted analysis and 0.14 mg/L (95% CI = 0.03–0.25) in fully adjusted model.	A strong and statistically robust positive association between presence of depressive symptoms and CRP levels.
[71]	Cross-sectional study (Republic of Korea)	Association between CRP levels and depressive symptoms in an elderly Korean population	569 (M = 224 F = 345) recruited from Korean Social Life, Health and Aging Project Health Examination Cohort aged 60 or over	Blood CRP levels CESD	CRP levels had significant associations with depressive symptoms before (β = 0.420, *p* = 0.010) and after (β = 0.336, *p* = 0.025) adjusting for age, BMI, systolic blood pressure, number of comorbidities, smoking status, alcohol intake, marital status, education, and sleep duration In women, the association between CRP levels and depressive symptoms was not significant before (*p* = 0.250) and after (*p* = 0.256) adjustment	Elevated CRP levels are independently associated with the presence of depressive symptoms in elderly Korean men
[72]	Cross-sectional study (Iran)	Association between serum hs-CRP levels and depression score in adolescent girls	563 adolescent girls aged 12–18 years	BDI-II hs-CRP levels	Serum hs-CRP was 0.61 (0.30–0.88) mg/L in the non-depressed group, 0.97 (0.50–1.82) mg/L in the group with a mild depression score, 1.04 (0.57–1.60) mg/L in those with a moderate depression score, and 0.84 (0.45–2.64) mg/L in girls with severe depression (Kruskal-Wallis test, *p* < 0.001) showing that hs-CRP is significantly higher in depressed groups Multinomial logistic regression analysis, controlling for age, BMI, waist circumference, social class, alcohol consumption, smoking or being passive smoker, and recent infections, showed that depression scores were positively associated with serum hs-CRP level (OR = 1.93, *p* < 0.001) Using a linear model after adjustment, B (the unstandardized beta) of hs-CRP according to the depression score was 1.43 (*p* < 0.001)	There is a significant association between serum hs-CRP levels and depression score in adolescent girls
[73]	Cross-sectional study (USA)	To find a possible association between inflammation and specific depressive symptoms	5909 patients recruited from ELSA	CES-D blood CRP levels	CRP levels were associated with symptoms of fatigue (*p* < 0.001), restless sleep (*p* = 0.03), low energy (*p* = 0.02) and feeling depressed (*p* = 0.04) These associations disappeared in patients under antidepressant medication	CRP levels are associated with specific symptoms and are modified by antidepressant
[74]	Cross-sectional study (Poland)	To determine whether there are differences in CRP levels between different psychiatric disorder	458 schizophrenia patients 319 unipolar depression 146 BD 114 BD depression 32 BD mania	ICD-10 serum CRP levels	No differences were observed in CRP levels between different psychiatric disorders	More than 1/3 of unipolar depression had high levels of CRP (i.e., > 3 mg/L)
[75]	Cross-sectional study (Sri Lanka)	To consider the extent to which shared genetic and environmental factors may contribute to the association between CRP levels and depression	2577 twins and 899 singletons	BDI-II blood hs-CRP levels Structural Equation Modelling	CRP levels were significantly associated with BMI (*p* < 0.01) but not depression (*p* > 0.05) In males, variance in CRP levels was explained by shared environment (51%; 95% CI = 13–62) and non-shared environment (45%; 95% CI = 36–54). In females, CRP variance was explained by genetic (41%; 95% CI = 10–52) and non-shared environment (56%; 95% CI = 47–67). A genetic association was found between CRP levels and BMI in females	No association between CRP levels and depression was found Inflammation might contribute to the development of some, but not all types of depression
[76]	Cross-sectional study (China)	Possible associations between CRP levels and depressive symptoms among the middle-aged and elderly in China	9459 Chinese middle-aged and elderly individuals (M = 4404F = 5055 selected on the CHARLS	CES-D blood CRP levels	No statistically significant associations were found between CRP levels and depressive symptoms in both Chinese middle-aged and elderly men and women	No association was found between CRP levels and depression
[77]	Cross-sectional study (UK)	Association between CRP levels and a worse response to escitalopram and better response to nortriptyline in consideration of genetic disposition to inflammation	755 unrelated individuals	MADRS GENDEP PRS for CRP	Higher PRS for CRP was associated with a better response to escitalopram and worse response to nortriptyline, reflected in a statistically significant interaction between polygenic risk score and drug (beta = 1.07; 95% CI = 0.26–1.87, *p* = 0.0093)	The association between CRP-PRS and antidepressant is in opposite direction of serum CRP measurement previously observed, which may be driven by state factors distinct from genetic influences on systemic inflammation
[78]	Case-control study (UK)	To explore CRP levels in MDD and its phenotypic associations	102 TRD patients with MDD currently experiencing depression, 48 treatment-responsive patients with MDD not currently experiencing depression, 48 patients with depression who were not receiving medication, and 54 healthy volunteers	Plasma CRP levels BMI Questionnaire assessments of depression, anxiety, and childhood trauma (no scihub)	CRP was elevated in patients with MDD, and more so in treatment-resistant patients Other phenotypes associated with elevated CRP included childhood adversity and specific depressive and anxious symptoms	Patients with MDD stratified for CRP might have a distinctive clinical profile that could be responsive to second-line treatment with anti-inflammatory drugs
[79]	Case-control study (China)	To examine whether CRP levels could be used to differentiate between MDD and BD II	96 healthy controls, 88 BD-II and 72 MDD drug-naïve patients in their major depressive episode	HDRS Plasma CRP levels	After treatment, CRP levels remained significantly different (*p* < 0.001), although HDRS scores were not significantly different between the BD-II and MDD patients A baseline CRP level of 621.6 ng/mL could discriminate between BD-II and MDD, with an area under the curve of 0.816 and a sensitivity and specificity of 0.699 and 0.882, respectively Baseline CRP level greater than 621.6 ng/mL had 28.2 higher ODs of a diagnosis of BD-II (*p* < 0.001, 95% CI = 10.96–72.35)	CRP may play a role as biomarker to differentiate between MDD and BD-II depression in both depressed and euthymic state
[80]	Case-control Study (UK)	To identify a distinct phenotypic profile of depression associated with inflammation	84 depressed patient divided in two group: with inflammation (CRP ≥ 3 mg/L) (N = 40) and without inflammation (CRP < 3 mg/L) (N = 44)	ICD-10 CIS-R BDI-II serum hs-CRP levels	The inflammation group had higher depression severity (adjusted mean difference = 8.82; 95% CI = 3.91–13.72; *p* = 0.03), somatic symptoms (adjusted mean difference = 3.25; 95% CI = 1.58–4.92; *p* = 0.02), perceived stress (adjusted mean difference = 4.58; 95% CI = 1.98–7.18; *p* = 0.02) and fatigue (adjusted mean difference = 9.71; 95% CI = 3.09–6.33; *p* = 0.02), but not with anhedonia. The inflammation group reported poorer quality of life (adjusted mean difference = −0.18; 95% CI = −0.32–0.05; *p* = 0.02) Regarding depressive symptoms, the inflammation group had increased guilty feelings (OR = 7.28; 95% CI = 2.09–31.17), pessimism (OR = 5.38; 95%CI = 1.53–22.73), concentration difficulties (OR = 4.56: 95% CI = 1.53–19.02) and indecisiveness (OR = 4.21; 95% CI = 1.15–18.45) compared to not inflammation group	A positive correlation was found between inflammation and both psychological and somatic symptoms of depression
[81]	Case-control study (India)	To compare CRP levels in late-onset depression compared with age-matched healthy controls and evaluate whether (any) association between CRP levels and depressive symptoms severity	25 patients aged ≥ 55 years with a first depressive episode and 27 age matched healthy controls	ICD-10 HDRS serum hs-CRP	Higher CRP levels were found in subjects with a current depressive episode compared with healthy controls (*p* = 0.001) CRP levels were strongly and positively associated with depression severity (r = 0.935; *p* < 0.001)	CRP may be related to late onset depression
[82]	Case-control study (USA)	To examine CRP levels in depressive disorders and evaluate the impact of SSRI	A two-part study:1–32 patients with history of depression (20 currently depressed, 12 euthymic) treated with SSRI and 20 healthy comparison group2-CRP measured in 20 MDD patients both before and after SSRI treatment	DSM-IV CRP levels	Study 1: no differences between CRP levels was observed in all the groups Study 2: CRP levels decreased significantly following SSRI treatment	Antidepressant may induce an anti-inflammatory response independently of antidepressant action
[83]	Case-control study (UK)	To assess the inflammation in MDD subjects through CRP levels and the possible association with genetic, lifestyle, and phenotypic factors	26,894 MDD patients and 59,000 healthy controls	CIDI plasma CRP levels	CRP levels were significantly higher in MDD patients than in the control group (*p* < 0.001) More MDD patients compared to healthy controls displayed CRP levels > 3 mg/L (21.2% compared with 16.8%, respectively) More healthy controls than MDD patients displayed CRP levels < 1 mg/L (47.0% compared with 42.6%, respectively) The polygenic risk score for MDD was significantly associated with log CRP levels, but this association was no longer significant after adjustment for covariates	CRP levels are increased in MDD patients independently by confounders
[84]	Case-control study (China)	Correlation of clinical features with hs-CRP levels in TRD patients	103 TRD and 103 non-TRD patients	HAMD-17 Plasma CRP levels	In TRD group, the course of disease was longer, the onset was earlier and the educational level was lower than that in the non-TRD group HAMD score (r = 0.338, *p* = 0.031), anxiety/somatization factor score (r = 0.465, *p* = 0.015) and sleep disorder (r = 0.387, *p* = 0.029) of TRD patients were positively correlated with the hs-CRP levels The onset age (r = −0.59, *p* = 0.009) was negatively correlated with the hs-CRP level	Positive association between hs-CRP levels and depressive symptoms in TRD individuals
[85]	Case-control study (China)	Considering CRP SNPs could regulate plasma CRP levels, the study hypothesized that inherited CRP allelic variations may covary with depressive symptomatology	60 depression patients with family depression history and 60 healthy control volunteers	CRP blood levels Genome CRP SNPs	A significantly higher circulating CRP level was found in patients with a positive family history Some inherited CRP SNPs (A allele in rs1417938 and C allele in rs1205) could up regulate serum CRP level and be distributed more in depression patients with family history	Genetically increased serum CRP level through SNPs variation is likely to induce family inherited depression
[86]	Case-control study (Poland)	To determine differences regarding CRP levels between elderly patients with unipolar depression and healthy controls	404 patients (202 with unipolar depression202 healthy controls)	serum CRP levels	No significant differences were observed between CRP level in the study groups (*p* = 0.96) CRP levels was not associated with age (*p* = 0.10) About 30% of the sample showed CRP levels > 3 mg/L	No association was found between CRP levels and depression in elderly patients
[87]	Case-control study (China)	To investigate whether inherited CRP allelic variations may co-vary with depressive symptoms	200 patients (100 MDD, with or without family depression history and 100 healthy controls)	CRP blood levels Genome CRP SNPs	Significantly higher circulating CRP levels were found in patients with a positive family history Certain inherited CRP SNPs (A allele in rs1417938 and C allele in rs1205) could up-regulate serum CRP levels and thus be associated with depression occurrence	Patients with a positive family history have higher CRP blood levels Certain inherited CRP SNPs could up-regulate serum CRP levels associated with depression occurrence
[88]	Cohort study	Association between CRP levels and depressive symptomatology among older adults	3397 participants from the English Longitudinal Study of Ageing	Blood CRP levels 8-item CESD	Baseline high CRP levels were associated with subsequent elevated symptoms of depression (OR = 1.49; 95% CI = 1.19–1.88). This relationship was no longer significant after simultaneous adjustments for metabolic and health variables After adjusting for baseline CRP levels, baseline elevated depressive symptoms were not associated with subsequent high CRP levels (OR = 1.12; 95% CI = 0.88–1.42)	Association between high CRP levels and elevated depressive symptomatology is determined by clinical factors
[89]	Cohort study(USA)	To compare the effect of current depression with the effect of cumulative episodes of depression on the CRP levels	1334 children, adolescents, and young adults	CAPA/YAPA serum hs-CRP levels	CRP levels at baseline were not associated with later depression status. Cumulative depressive episodes predicted later CRP levels	CRP levels were increased by the occurrence of multiple depressive episodes
[40]	Cohort study (Brazil)	To evaluate the association between persistent depressive symptoms and the onset of low-grade inflammation	1508 young individuals (134 with persistent depressive symptoms and 1374 negative at BDI)	BDI plasma hs-CRP levels	Low-grade inflammation (CRP > 3 mg/L) was more frequently observed in BDI+ group compared to the BDI-group (20.9% vs 11.4%, *p* = 0.001) After adjusting for sex, age, waist circumference, BMI, levels of physical activity, smoking and prevalence of metabolic syndrome, persistent depressive symptoms remained an independent predictor of low-grade inflammation onset (OR = 1.76; 95% CI = 1.03–3.02; *p* = 0.04)	Persistent depressive symptomatology was independently associated with low-grade inflammation (CRP levels) onset among healthy individuals
[90]	Cohort study (USA)	Association between depression and hs-CRP levels	508 healthy adults (F = 49%, mean age 48.5 yy)	BDI-II Average serum hs-CRP levels	Individuals with higher depression scores have higher levels of hs-CRP An independent association was observed in women Body mass index appears to be a partial mediator of this association	A positive association was found between hs-CRP levels and depression score in women
[91]	Cohort study (The Netherlands)	To assess whether depression and apathy had different etiologiesin the elderly	599 elderly subjects assessed annually form age 85 to 90	MMSE GDS-15 hs-CRP levels	At baseline no association was found between CRP levels and apathy or depression Subjects with highest CRP levels at baseline had significantly more depressive symptoms during follow-up	Higher CRP concentration increased the risk of depression but not apathy in elderly subjects
[92]	Cohort study (USA)	Association between depressive symptoms and CRP levels in mid-life women	3302 pre- and early perimenopausal women	Blood CRP levels CESD	Higher CESD scores predict higher subsequent CRP levels and vice versa over a 7-year period Higher CRP levels at year X predicted higher CES-D scores at year X+1 (*p* = 0.03)	Bi-directional association was found between depressive symptoms and CRP levels in mid-life women
[29]	Cohort Study (UK)	To evaluate if increasing levels of CRP in childhood and/or early-adulthood is associated with the risk of depression in early-adulthood	1561 participants (M = 770; F = 791)	CIS-R ICD-10 criteria serum hs-CRP levels measured at age 9, 15 and 18	Subjects with persistently low hs-CRP levels (N = 463; 30%) showed the lowest average CRP values at all ages. Subjects in the decreasing group (N = 360; 23%) showed the second highest CRP values at 9 and 15 years of age, which decreased to the second lowest value at 18. Subjects in the increasing group (N = 367; 24%) showed the second lowest CRP values at 9 and 15 years, which increased to the highest levels at 18 years, with a not statistically significant higher risk of developing depression at 18 years. Adjusted OR = 1.33 (95% CI = 0.73–2.39). Subjects with persistently high hs-CRP levels (N = 371; 24%) showed the highest CRP values at 9 and 15 years, and the second highest at 18 year., with a not statistically significant higher risk of developing depression at 18 years. Adjusted OR = 2.54 (95% CI = 0.90–7.16). Subjects in the increasing sample that showed increases in CRP over the years had an increased risk of developing moderate/severe depression at age 18 (OR = 3,78, 95%; CI = 1.46–9.81; *p* = 0.006)	An increasing CRP levels pattern from adolescence to early-adulthood is associated with the risk of developing depression in the early-adulthood
[93]	Cohort study (Australia)	Association between CRP levels and increased risk of *de novo* MDD	1494 randomly selected women	Serum hs-CRP levels SCID-I/NP	The hazard ratio for depression increased by 44% for each standard deviation increases in log-transformed hs-CRP (ln-hsCRP) (HR = 1.44, 95% CI = 1.04–1.99), after covariates adjusting	hs-CRP levels is an independent risk marker for *de novo* MDD
[94]	Cohort Study	To examine long-term patterns of systemic inflammation in aging adults and determined whether individuals with chronic elevations in inflammation were at increased risk for having symptoms of depression as older adults	4476 participants (mean age: 75.5(SD = 5.1)) M = 1775; F = 2701	hs-CRP levels measured during a 21-year period in three moments CESD	Individuals who maintained elevated CRP levels at two of three visits (“unstable elevated”; ß = 0.09; 95% CI = 0.02–0.17; *p* = 0.019) and participants who maintained elevated CRP at all three visits (“stable elevated”; ß = 0.13; 95% CI = 0.05–0.21; *p* = 0.002) had significantly greater depressive symptoms during late-life, after adjusting for demographic characteristics and cardiovascular risk factors Subjects with “stable elevated” 21-year CRP pattern displayed higher depressive symptomatology during late-life (ß = 0.12; 95% CI = 0.03–0.21, *p* = 0.012) Subjects with “unstable elevated” and “stable elevated” 21-year CRP patterns were significantly associated with greater somatic depression symptomatology, but not with affective or interpersonal depression symptomatology	Subjects with “stable elevated” CRP levels s associated with higher risk of developing late-life depression
[95]	Cohort study (China)	To test whether baseline serum CRP levels could predict antidepressant treatment responses	75 adult inpatients (M = 26 F = 49) with major MDD	HDRS-17 Plasma CRP levels	The two groups differed in HDRS-17 scores at week 4 (*p* = 0.012), with the low CRP group having lower HDRS-17 scores than the high CRP group The low CRP group exhibited higher percent reduction in HDRS-17 scores at week 3 (*p* = 0.028) and week 4 (*p* = 0.003) as compared to the high CRP group The remission rate was higher in the low CRP group (*p* = 0.010)	Baseline serum CRP levels may predict antidepressant treatment responses in patients with MDD Patients with higher levels of CRP were less likely to get remission
[96]	RCT (Australia)	To measure the efficacy of anti-inflammatory augmentation of antidepressant treatment in MDD patients and whether treatment response was dependent on baseline inflammation levels	119 MDD	Diagnosis of current major depression based on the MINI and according to DSM-IV criteria Plasma CRP levels MADRS THINK-it FAST	There was no evidence of superior efficacy of celecoxib augmentation over placebo There was no evidence that pre-treatment inflammation levels modified the effect of celecoxib augmentation versus placebo	CRP may not be suitable to predict treatment selection and response in MDD
[97]	RCT (USA)	To evaluate the sex differences in the association between CRP levels and the response to antidepressant treatments	220 individuals (M = 75 F = 145) from EMBARC study	HAMD-17 plasma CRP levels	Significant sex differences in association of baseline-to-week-8 HAMD-17 reduction with baseline CRP levels (*p* = 0.033) Higher baseline CRP levels were associated with lower baseline-to-week-8 HAMD-17 reduction in females (*p* < 0.0001) but not in males (*p* = 0.632)	Elevated baseline CRP levels were associated with worse antidepressant treatment outcomes in females
[98]	RCT	To test the hypothesis that CRP predicts differential response to escitalopram and nortriptyline	241 MDD	MADRS CRP levels	CRP levels at baseline differentially predicted treatment outcome with the two antidepressants (CRP-drug interaction: β = 3.27, 95% CI = 1.65–4.89) For patients with low levels of CRP (<1 mg/L), improvement on the MADRS score was 3 points higher with escitalopram For patients with higher CRP levels, improvement on the MADRS score was 3 points higher with nortriptyline CRP and its interaction with medication explained more than 10% of individual-level variance in treatment outcome	Higher CRP levels are associated with a better response to nortriptyline than escitalopram

BD = bipolar disorder; BDI-II = Beck Depression Inventory; CAPA = Child and Adolescent Psychiatric Assessment; CESD = Center for Epidemiological Studies-Depression scale; CIS-R = Clinical Interview Schedule, Revised; CHARLS = Chine Health and Retirement Longitudinal Study; CIDI = Composite International Diagnostic Interview; CPT = Continuous Performance Test; DSM-IV = Diagnostic and Statistical Manual of Mental Disorders-IV; ELSA = Longitudinal Study of Adult HealthEMARC = Establishing Moderators and Biosignature of Antidepressant Response for Clinical Care; FAST = Functioning Assessment Short Test; FTT = Finger-Tapping Test; GDS-15 = 15-item Geriatric Depression Scale; GENDEP = Genome-Based Therapeutic Drugs for Depression; GHQ = General Health Questionnaire score; HAMD17 = HDRS-17 = 17-item Hamilton Rating Scale for Depression 17; HARS = Hamilton Anxiety Rating Scale; hs-CRP = high sensitivity C-reactive protein; HSCL-25 = Hopkins Symptom Checklist-25; ICD-10 = International Classification of Disease 10th Revision; MADRS = Montgomery-Åsberg Depression Rating Scale scores; MDD = Major depressive disorder; MINI = Mini-International Neuropsychiatric Interview; MMSE = Mini Mental State Examination; NHANES = National Health and Nutrition Examination Survey; OCD = obsessive compulsive disorder; PCS = physical component summary score of the SF-36 Health Survey; PDS = Psychological Distress Scale; PHQ-9: Patient health Questionnaire-9; PRS = Polygenic risk score; RCT = Randomized controlled trial; RFFT = Ruff Figural Fluency Test; SCID-I/NP = Structured Clinical Interview nonpatient version; SHAPS = Snaith-Hamilton Pleasure Scale; SNPs = single-nucleotide polymorphisms; SSRI = Selective Serotonin Reuptake Inhibitor; SZSRDS = Short Zung Self Rating Depression Scale; THINC-It = THINC-Integrated Tool; TRD = treatment resistant depression; WCST = Wisconsin Card-Sorting Test; YAPA = Young and Adult Psychiatric Assessment.

**Table 2:** Quality of the included studies, based on modified version of the Newcastle-Ottawa Quality Assessment Scale, adapted for cross-sectional studies.

Study	Selection				Comparability	Outcome		Overall
	Representativeness of the sample	Sample size	Non-respondents	Ascertainment of depression	Based on design and analysis	Assessment of the outcome	Statistical test	
[70]	*	/	/	*	*	**	*	6
[61]	*	*	/	*	**	**	*	8
[52]	*	/	*	*	**	**	*	8
[58]	*	/	/	**	**	**	*	7
[47]	/	/	/	*	**	**	*	6
[59]	*	/	/	**	*	**	*	7
[74]	*	/	/	*	*	**	*	6
[49]	*	/	/	*	**	**	*	7
[50]	/	/	*	*	/	**	*	5
[68]	*	/	/	*	**	**	*	7
[76]	*	/	*	*	**	**	*	8
[62]	*	*	/	*	**	**	*	8
[75]	*	/	/	**	**	**	*	8
[54]	*	*	/	*	**	**	*	8
[64]	*	*	/	*	**	**	*	8
[71]	*	/	/	*	**	**	*	7
[55]	*	*	/	*	**	**	*	8
[66]	*	*	*	*	**	**	*	9
[67]	*	/	/	*	*	**	*	6
[63]	*	/	/	*	**	**	*	7
[72]	*	/	/	*	**	**	*	7
[51]	*	*	/	*	**	**	*	7
[48]	*	/	/	*	**	**	*	7
[73]	*	/	/	*	**	**	*	7
[69]	*	/	/	*	/	**	*	5
[56]	*	*	/	*	**	**	*	9
[57]	*	/	/	*	**	**	*	7
[65]	*	/	/	*	**	**	*	7
[45]	*	/	/	*	**	**	*	7
[46]	*	/	/	*	**	**	*	7
[60]	*	*	/	*	**	**	*	8
[53]	*	/	/	*	**	**	*	7
[77]	*	/	/	*	*	**	*	6

Note: /: 0 point; *: 1 point; **: 2 points.

**Table 3:** Quality of the included studies, based on modified version of the Newcastle-Ottawa Quality Assessment Scale, adapted for case-control studies.

Study	Selection				Comparability	Exposure			Overall
	Is the case definition adequate?	Representativeness of the case	Selection of controls	Definition of controls	Based on design or analysis	Ascertainment of exposure	Same method ascertainment	Non- response rate	
[80]	*	*	/	/	*	*	*	/	5
[83]	*	*	*	*	**	*	*	/	8
[81]	*	*	*	*	*	*	*	/	7
[79]	*	/	*	/	*	*	*	/	5
[86]	*	/	*	*	*	*	*	/	6
[87]	*	/	/	*	*	*	*	/	5
[85]	*	/	*	*	*	*	*	/	6
[84]	*	*	*	/	*	*	*	/	6
[78]	*	/	*	*	**	*	*	/	7
[82]	*	/	/	*	*	/	*	/	5

Note: /: 0 point; *: 1 point; **: 2 points.

**Table 4:** Quality of the included studies, based on modified version of the Newcastle-Ottawa Quality Assessment Scale, adapted for cohort studies.

Study	Selection				Comparability	Outcome			Overall
	Representativeness of the exposed cohort	Selection of the non-exposed cohort	Ascertainment of exposure	Outcome was not present at start	Based on design and analysis	Assessment of outcome	Enough follow-up	Adequacy of follow-up	
[29]	*	*	*	/	**	*	*	*	8
[94]	*	*	*	/	**	*	*	*	8
[100]	/	*	*	*	**	*	*	/	7
[89]	/	*	*	/	**	*	*	*	7
[88]	*	*	*	/	**	*	*	*	8
[90]	/	*	*	/	**	*	*	*	7
[92]	*	*	*	/	**	*	*	*	8
[93]	*	*	*	/	**	*	*	*	8
[91]	*	*	*	/	/	+	*	*	6
[95]	/	*	*	/	*	*	/	*	5

Note: /: 0 point; *: 1 point; **: 2 points.

**Table 5:** Quality of the included studies, based on modified version of the Newcastle-Ottawa Quality Assessment Scale, adapted for RCT.

Study	Selection				Comparability	Outcome			Overall
	Is the case definition adequate?	Representativeness of the case	Selection of control	Definition of control	Based on design and analysis	Assessment of exposure	Same method ascertainment	Non- response rate	
[97]	*	*	*	*	**	*	*	/	8
[98]	*	*	*	*	*	*	*	/	7
[96]	*	*	*	*	*	*	*	/	7

Note: /: 0 point; *: 1 point; **: 2 points.

## Data Availability

Not applicable.

## Supplementary Materials

The following supporting information can be downloaded at: https://www.mdpi.com/article/10.3390/ijms23031616/s1.
