# Supplementary material for: C-Reactive Protein as a Biomarker for Major Depressive Disorder?
Source: Int J Mol Sci. 2022 Jan 30;23(3):1616. doi: 10.3390/ijms23031616 (PMC8836046; doi:10.3390/ijms23031616)
Supplement: Supplementary file 1 [file ijms-23-01616-s001.zip › ijms-1561066-Supplementary-Newcastle-OTTAWA quality assessment scale.pdf]

International Journal of *Molecular Sciences*

MDPI

Table S1. New Castle-OTTAWA Quality Assessment Scale: Cross-sectional Studies.

**Selection:** (Maximum 5 stars)	1) Representativeness of the sample:	a) Truly representative of the average in the target population. * (all subjects or random sampling)
		b) Somewhat representative of the average in the target population. * (non- random sampling)
		c) Selected group of users.
		d) No description of the sampling strategy.
	2) Sample size:	a) Justified and satisfactory.
		b) Not justified.
	3) Non-respondents:	a) Comparability between respondents and non-respondents characteristics is established, and the response rate is satis-factory. *
		b) The response rate is unsatisfactory, or the comparability between respondents and non-respondents is unsatisfactory.
		c) No description of the response rate or the characteristics of the responders and the non-responders.
	4) Ascertainment of the exposure:	a) Validated measurement tool. **
		b) Non-validated measurement tool, but the tool is available or described.*
		c) No description of the measurement tool.
**Comparability:** (Maximum 2 stars)	**1)** The subjects in different outcome groups are comparable, based on the study design or analysis. Confounding factors are con-trolled:	a) The study controls for the most important factor (select one). *
		b) The study control for any additional factor. *
**Outcome:** (Maximum 3 stars)	**1)** Assessment of the outcome:	a) Independent blind assessment. **
		b) Record linkage. **
		c) Self report. *
		d) No description.
	**2)** Statistical test:	a) The statistical test used to analyze the data is clearly described and appropriate, and the measurement of the association is presented, including confidence intervals and the probability level (p value). *
		b) The statistical test is not appropriate, not described or incomplete.

*: 1 point; **: 2 points.

Table S2. New Castle-OTTAWA Quality Assessment Scale: Case Control Studies.

**Selection**	1) Is the case definition adequate?	a) yes, with independent validation *
		b) yes, eg record linkage or based on self reports
		c) no description
	2) Representativeness of the cases	a) consecutive or obviously representative series of cases *
		b) potential for selection biases or not stated
	3) Selection of Controls	a) community controls *
		b) hospital controls
		c) no description
	4) Definition of Controls	a) no history of disease (endpoint) *
		b) no description of source
**Comparability**	1) Comparability of cases and controls on the basis of the design or analysis	a) study controls for__________(Select the most important factor.) *
		b) study controls for any additional factor * (This criteria could be modified to indicate specific control for a second important factor.)
**Exposure**	1) Ascertainment of exposure	a) secure record (eg surgical records) *
		b) structured interview where blind to case/control status *
		c) interview not blinded to case/control status
		d) written self report or medical record only
		e) no description
	2) Same method of ascertainment for cases and controls	a) yes *
		b) no
	3) Non-Response rate	a) same rate for both groups *
		b) non respondents described
		c) rate different and no designation

Note: A study can be awarded a maximum of one star for each numbered item within the Selection and Exposure categories. A maximum of two stars can be given for Comparability. *: 1 point

Table S3. New Castle-OTTAWA Quality Assessment Scale: Cohort Studies.

**Selection**	1) Representativeness of the exposed cohort	a) truly representative of the average _____(describe) in the community *
		b) somewhat representative of the average _____in the community *
		c) selected group of users eg nurses, volunteers
		d) no description of the derivation of the cohort
	2) Selection of the non exposed cohort	a) drawn from the same community as the exposed cohort *
		b) drawn from a different source
		c) no description of the derivation of the non exposed cohort
	3) Ascertainment of exposure	a) secure record (eg surgical records) *
		b) structured interview *
		c) written self report
		d) no description
	4) Demonstration that outcome of interest was not present at start of study	a) yes *
		b) no
**Comparability**	1) Comparability of cohorts on the basis of the design or analysis	a) study controls for__________(select the most important factor) *
		b) study controls for any additional factor * (This criteria could be modified to indicate specific control for a second important factor.)
**Outcome**	**1)** Assessment of outcome	a) independent blind assessment *
		b) record linkage *
		c) self report
		d) no description
	2) Was follow-up long enough for outcomes to occur	a) yes (select an adequate follow up period for outcome of interest) *
		b) no
	3) Adequacy of follow up of cohorts	a) complete follow up - all subjects accounted for *
		b) subjects lost to follow up unlikely to introduce bias - small number lost - > _____% (select an adequate %) follow up, or description provided of those lost) *
		c) follow up rate <______% (select an adequate %) and no description of those lost
		d) no statement

Note: A study can be awarded a maximum of one star for each numbered item within the Selection andOutcome categories. A maximum of two stars can be given for Comparability. *: 1 point

Table S4. New Castle-OTTAWA Quality Assessment Scale: Randomized Controlled Trial.

**Selection**	1) Is the case definition adequate?	a) yes, with independent validation *
		b) yes, e.g., record linkage or based on self reports
		c) no description
	2) Representativeness of the cases	a) consecutive or obviously representative series of cases *
		b) potential for selection biases or not stated
	3) Selection of Controls	a) community controls *
		b) hospital controls
		c) no description
	4) Definition of Controls	a) no history of disease (endpoint) *
		b) no description of source
**Comparability**	1) Comparability of cases and controls on the basis of the design or analysis	a) study controls for_____(Select the most important factor.) *
		b) study controls for any additional factor * (This criteria could be modified to indicate specific control for a second important factor.)
**Exposure**	1) Ascertainment of exposure	a) secure record (eg surgical records) *
		b) structured interview where blind to case/control status *
		c) interview not blinded to case/control status
		d) written self report or medical record only
		e) no description
	2) Same method of ascertainment for cases and controls	a) yes *
		b) no
	3) Non-Response rate	a) same rate for both groups *
		b) non respondents described
		c) rate different and no designation

*: 1 point
